# Tolerance in TCR/Cognate Antigen Double-Transgenic Mice Mediated by Incomplete Thymic Deletion and Peripheral Receptor Downregulation

**DOI:** 10.1155/1995/54219

**Published:** 1995

**Authors:** Clio Mamalaki, Marianna Murdjeva, Mauro Tolaini, Trisha Norton, Phillip Chandler, Alain Townsend, Elizabeth Simpson, Dimitris Kioussis

**Affiliations:** 1 lnstitute of Molecular Biology and Biotechnology Crete Greece; 2 Division of Molecular Immunology National Institute for Medical Research The Ridgeway Mill Hill London NW7 1AA United Kingdom; 3 MRC Clinical Sciences Centre Royal Postgraduate Medical School Hammersmith Hospital London W12 ONN United Kingdom; 4 Institute of Molecular Medicine John Radcliffe Hospital Headington Oxford OX3 9DU United Kingdom

**Keywords:** Tolerance, deletion, F5 TCR, nucleoprotein, double-transgenic mice

## Abstract

Influenza nucleoprotein (NP)-specific T-cell receptor transgenic mice (F5) were crossed with transgenic mice expressing the cognate antigenic protein under the control of the H- 2K^b^ promoter. Double-transgenic mice show negative selection of thymocytes at the CD4^+^8^+^TCR^10^ to CD4^+^8^+^TCR^hi^ transition stage. A few CD8 T cells, however, escape clonal deletion, and in the peripheral lymphoid organs of these mice, they exhibit low levels of the transgenic receptor and upregulated levels of the CD44 memory marker. Such cells do not proliferate upon exposure to antigen stimulation *in vivo* or *ex vivo*, however, they can develop low but detectable levels of antigen-specific cytotoxic function after stimulation *in vitro* in the presence of IL-2.


*Developmental Immunology*, 1996, Vol 4, pp. 299–315
Reprints available directly from the publisher
Photocopying permitted by license only

© 1996 OPA (Overseas Publishers Association)
Amsterdam B.V. Published in The Netherlands
by Harwood Academic Publishers GmbH
Printed in Singapore

### Tolerance in TCR/Cognate Antigen Double-Transgenic Mice Mediated by Incomplete Thymic Deletion and Peripheral Receptor Downregulation

CLIO MAMALAKI,[†] MARIANNA MURDJEVA,[‡] MAURO TOLAINI,[‡] TRISHA NORTON,[‡] PHILLIP CHANDLER,[§] ALAIN TOWNSEND,[||] ELIZABETH SIMPSON,[§] AND DIMITRIS KIOUSSIS[‡]

[†]*Institute of Molecular Biology and Biotechnology, Crete, Greece*
[‡]*Division of Molecular Immunology, National Institute for Medical Research, The Ridgeway, Mill Hill, London NW7 1AA, UK*
[§]*MRC Clinical Sciences Centre, Royal Postgraduate Medical School, Hammersmith Hospital, London W12 ONN, UK*
[||]*Institute of Molecular Medicine, John Radcliffe Hospital, Headington, Oxford OX3 9DU, UK*

Influenza nucleoprotein (NP)-specific T-cell receptor transgenic mice (F5) were crossed with transgenic mice expressing the cognate antigenic protein under the control of the H-$2K^b$ promoter. Double-transgenic mice show negative selection of thymocytes at the $CD4^+8^+TCR^{lo}$ to $CD4^+8^+TCR^{hi}$ transition stage. A few $CD8^+$ T cells, however, escape clonal deletion, and in the peripheral lymphoid organs of these mice, they exhibit low levels of the transgenic receptor and upregulated levels of the CD44 memory marker. Such cells do not proliferate upon exposure to antigen stimulation *in vivo* or *ex vivo*, however, they can develop low but detectable levels of antigen-specific cytotoxic function after stimulation *in vitro* in the presence of IL-2.

KEYWORDS: Tolerance, deletion, F5 TCR, nucleoprotein, double-transgenic mice.

### INTRODUCTION

Achieving and maintaining self-tolerance are of central importance for an effective immune system. In the case of T-cell tolerance, this is generally accomplished by elimination of self-reactive cells either during development in the thymus (Kappler et al., 1987; MacDonald et al., 1988) or after encountering self-antigen in the periphery (Jones et al., 1990; Webb et al., 1990; Kawabe and Ochi, 1991; Rocha et al., 1992); however, some potentially autoreactive cells are not physically deleted but are rendered unresponsive to self-antigens (Schwartz, 1989; Blackman et al., 1990; Ramsdell and Fowlkes, 1990). Such cells can be permanently incapacitated in their ability to respond to antigen or can be restimulated *in vitro* or *in vivo* by exposure to high levels of antigen and/or to different cytokines. The unresponsiveness maintained in some nondeletional tolerant states has been attributed to the downregulation of the reactive TCR (Schönrich et al., 1991) and/or the coreceptor CD4 or CD8 (Rocha et al., 1992). In addition, other factors such as transcription levels of certain genes or efficiency in costimulation and signal transduction may be affected in this process (Mueller et al., 1989).

It is important to understand the mechanisms underlying the induction and maintenance of the tolerant state of T cells in order to be able to intervene in cases where their untimely activation causes autoimmune disease. To study the phenomenon in more controlled situations, several groups have followed a strategy that attempts to keep one or more of the participating elements constant. Such central candidates are the T-cell receptor (TCR) and the cognate antigen. Thus, T-cell-receptor (TCR) transgenic mice have been crossed to mice that express the cognate antigen.

We have generated a TCR transgenic mouse that bears on most of its T cells a TCR (F5) that recognizes a nonamer peptide (αα366–374; NP peptide) from the influenza virus (A/NT/60/68) nucleoprotein in the context of class I MHC ($D^b$) (Townsend et al., 1986; Mamalaki et al., 1993a). Most T cells in

[‡]Corresponding author. National Institute for Medical Research, The Ridgeway, London NW7 1AA, UK.

F5 TCR transgenic mice are CD8+ cytotoxic cells and can respond to the cognate antigen (peptide or viral protein) both *in vivo* and *in vitro* (Mamalaki et al., 1992, 1993a). The utilization by the receptor of the $V\beta_{11}$ member of the β-chain gene family also confers reactivity to endogenous superantigens (Mtv 8, 9, and 11) when presented by H-2E class II MHC molecules (Dyson et al., 1991). In the past, we have reported the creation of a tolerant state in F5 TCR transgenic mice when they are crossed with H-2E+ mice carrying Mtv8 and Mtv9 (Mamalaki et al., 1993a). In that case, we demonstrated that hybrids of F5 mice bred with CBA (H-2$^k$) or BALB/c (H-2$^d$) mice lack double-positive thymocytes that express high levels of TCR and as a consequence a reduction in single-positive mature T-cells. CD4+ cells in the periphery of these mice are more severely affected in that there are fewer CD4+ cells expressing $V\beta_{11}$ compared to F5 H-2$^{bb}$ mice. A substantial number of CD8+ cells, however, with low levels of $V\beta_{11}$ transgenic chains accumulate in the peripheral lymphoid organs of F5/H2E+ mice. The circulating CD8+ cells from E+/Mtv8+9+ mice (even from those backcross animals homozygous for the H-2$^k$ haplotype) can be stimulated *in vitro* to differentiate into cytotoxic effector cells that can kill target cells in an NP-antigen-specific manner (Mamalaki et al., 1993a). This showed that T cells can be tolerant to one kind of antigen (endogenous superantigen in this case), but retain their ability to respond to nominal antigen (NP peptide).

To assess the development of F5 T cells and study the mechanisms of tolerance induction in transgenic mice in which the cognate antigen influenza nucleoprotein is a self-antigen, we generated transgenic mice that express the viral protein under the broadly active H-2K$^b$ promoter. Double-transgenic mice (F5TCR/H2NP) were assessed for F5 T-cell development and for their ability to respond to nucleoprotein antigen. Such mice appear tolerant mainly due to deletion of CD4+8+TCR$^{hi}$ thymocytes with the concomitant reduction in output of single-positive mature T cells. However, a small number of CD8+$V\beta_{11}^+$ cells appear in the periphery. These cells express lower levels of F5 TCR in comparison with T cells from F5 mice that do not express influenza nucleoprotein, they express high levels of CD44, and they are unable to respond to peptide antigenic stimulation *in vivo*. *In vitro*, they fail to proliferate in response to peptide, but can develop effector function after culture with the cytokine IL-2 for several days.

This system represents an experimental model of nondeletional tolerance that will allow the dissec-

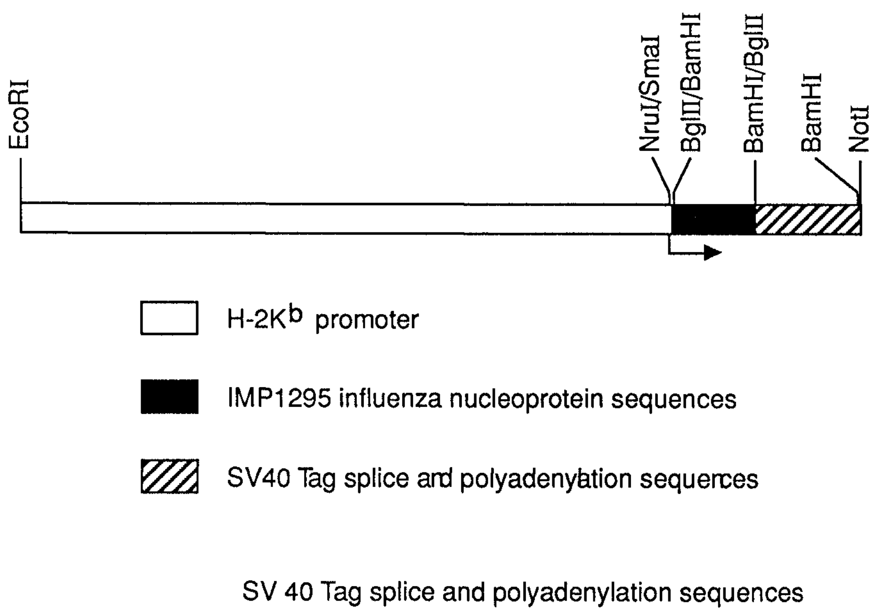

FIGURE 1. Influenza nucleoprotein expression construct used to generate H2NP transgenic mice.

tion of biochemical mechanisms of maintenance of tolerance as well as its potential reversal, which could lead to autoimmunity.

### RESULTS

### Generation of Influenza Nucleoprotein Transgenic Mice

In order to generate mice expressing a transgenic influenza nucleoprotein, we placed the expression of the transgene under the control of the widely expressed Class I MHC promoter H-2K$^b$ (H2NP) (Weiss et al., 1983). The gene for nucleoprotein in these constructs is a deletion mutant (IMP1295) of the full gene (Davey et al., 1985; Townsend et al., 1985). The deletion removes αα3 to 327 from the N′ terminal portion of the protein and probably renders the protein nonfunctional and, thus, less likely to be harmful when expressed in the transgenic mouse cells. However, it contains the part of the protein that gives rise to the epitope (αα366–374) recognized by the F5 TCR (Townsend et al., 1986). The H-2K$^b$ promoter was joined to the deleted NP gene, and an intron and poly-A signal from the SV40 T antigen were added to generate the H2NP construct (Fig. 1). In transgenic mice expressing this construct, the foreign antigen becomes a self-protein expressed in most cells of the body.

The construct was injected into fertilized mouse (C57B1/10) eggs and several transgenic lines were generated: four of these were used in this study (H2NP10, H2NP22, H2NP40, and H2NP47). The messenger RNA for the nucleoprotein proved to be too unstable to allow us to perform Northern analysis studies for expression. Polymerase chain reaction (PCR) on RNA from tissues of NP transgenic-mice assays, however, established that the transgene was expressed in these mice (data not shown).

### Antigen-Presenting Cells in H2NP Transgenic Mice Express and Present Influenza Nucleoprotein Peptides

In order to assess the level of expression of nucleoprotein by professional antigen-presenting cells (APC) in lymphoid organs of H2NP mice, macrophages and dendritic cells from the spleen or the thymus of these mice were isolated and used to stimulate F5 T cells. Figure 2 shows the proliferation of F5 T cells after incubation with H2$^{bb}$ (C57B1/10) APC, H2$^{bb}$ APC loaded with NP peptide, or with APC from H2NP40 and H2NP47 mice. The results show that peripheral (Fig. 2A) and thymic (Fig. 2B) antigen-presenting cells from H2NP40 mice can stimulate F5 T cells with similar efficiency as H2$^{bb}$ APC loaded with NP peptide. Similar results were obtained using APCs from H2NP22 mice (data not shown). This confirms expression of the NP transgene in APCs of these mice. Antigen-presenting cells from the spleen or thymus of H2NP47 mice, on the other hand, stimulated F5 T cells to a lesser extent but consistently above background. This indicates that expression of NP in H2NP47 transgenic mice is lower than that found in H2NP22 or H2NP40 mice.

### Generation of Double (F5/NP)-Transgenic Mice

To assess the effects of an antigenic molecule expressed as self-protein on the development of F5 T cells, the H2NP transgenic mice were crossed with the F5 TCR transgenic mice. F5/H2NP double-transgenic mice were analyzed by FACS analysis and their T-cell development was compared with that seen in F5(H-2$^b$) single-transgenic mice. The absolute numbers of thymocytes showed a tendency to be reduced in mice expressing transgenic nucleoprotein. For example, F5/H2NP10 double-transgenic mice showed approximately one-third to one-half the number of thymocytes seen in F5 control mice. Figure 3A shows thymocytes from double-transgenic mice stained for CD4 and CD8. In all double-transgenic mice, we observed that the proportion of CD4$^+$8$^+$ and the absolute numbers of CD4$^+$ cells were not affected, whereas the proportion of thymocytes that developed into fully mature CD8$^+$4$^-$ cells was reduced. The percentage numbers of Fig. 3A in the lower right quadrant includes cells with downregulated coreceptor levels (probably due to negative selection) and these numbers do not reflect genuine CD8$^+$ single-positive cells.

Three-color FACS analysis of F5 thymocytes stained with antibodies against CD4, CD8, and V$\beta_{11}$ normally shows two populations of cells with different levels of TCR (Fig. 3C): one that stains dull for TCR (TCR$^{lo}$) and represents the majority of double-positive immature thymocytes (Mamalaki et al., 1993a); and a brightly staining population (TCR$^{hi}$) that represents mainly single-positive mature T cells and those double-positive cells that have already upregulated their TCR, including the intermediate populations CD4$^+$8$^{lo}$ and CD4$^{lo}$8$^+$ (Mamalaki et al.,

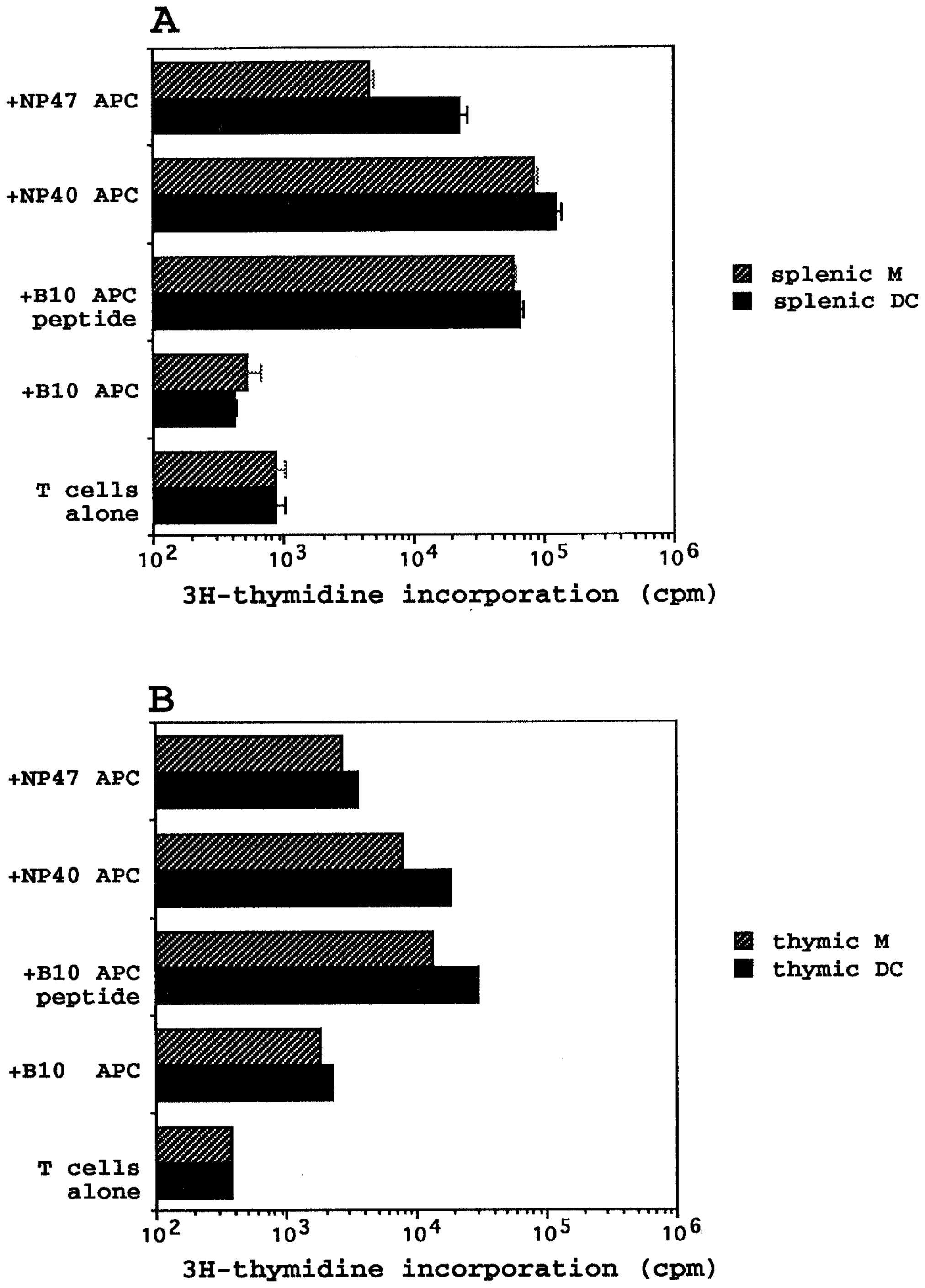

FIGURE 2. *In vitro* proliferation of F5/RAG$^{-/-}$ T cells in the presence of NP macrophages and dendritic cells. T cells from F5/RAG$^{-/-}$ spleens were stimulated *in vitro* by dendritic cells (DC) and macrophages (M) isolated from (A) spleens and (B) thymuses of NP40 and NP47 mice. B10 DC and M from the same tissues, loaded with the antigenic peptide beforehand and then washed, were used as a positive control. Proliferation of the responders was measured by $^3$H-thymidine incorporation.

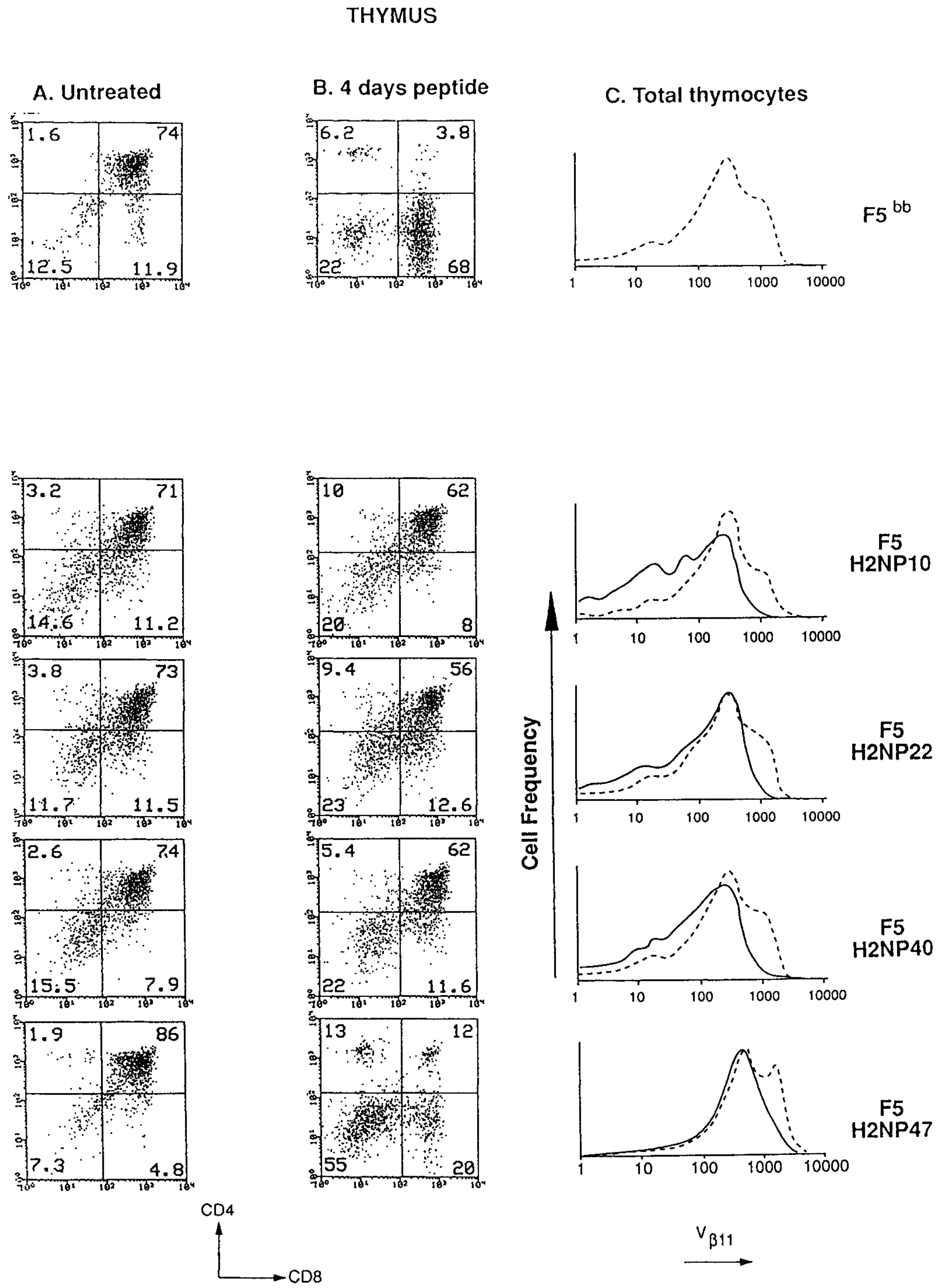

FIGURE 3. Absence of TCR+hi cells in the thymus of F5/H-2NP double-transgenic mice. Three color fluorometric analysis was performed on thymocytes of TCR single-transgenic mice or TCR/NP double-transgenic mice before (A) or after (B) intraperitoneal treatment for 4 days with 50 n moles of antigenic peptide. Cells were stained with anti-CD8 FITC, anti-CD4 PE, and biotinylated anti-V$\beta_{11}$ followed by Tricolor-conjugated streptavidin as described in Materials and Methods. Dot blots represent two-color analysis of cells stained with CD4 and CD8. Numbers represent percent proportion of cells in the quadrant. (C) Three-parameter data files were software-gated to generate single-color staining histograms of V$\beta_{11}$ expression on total thymocytes. Numbers indicate the mean fluorescence of V$\beta_{11}$ staining in arbitrary units. Solid line: V$\beta_{11}$ expression on total thymocytes from double-transgenic mice. Dashed line: V$\beta_{11}$ expression on total thymocytes from the control F5 single-transgenic mouse.

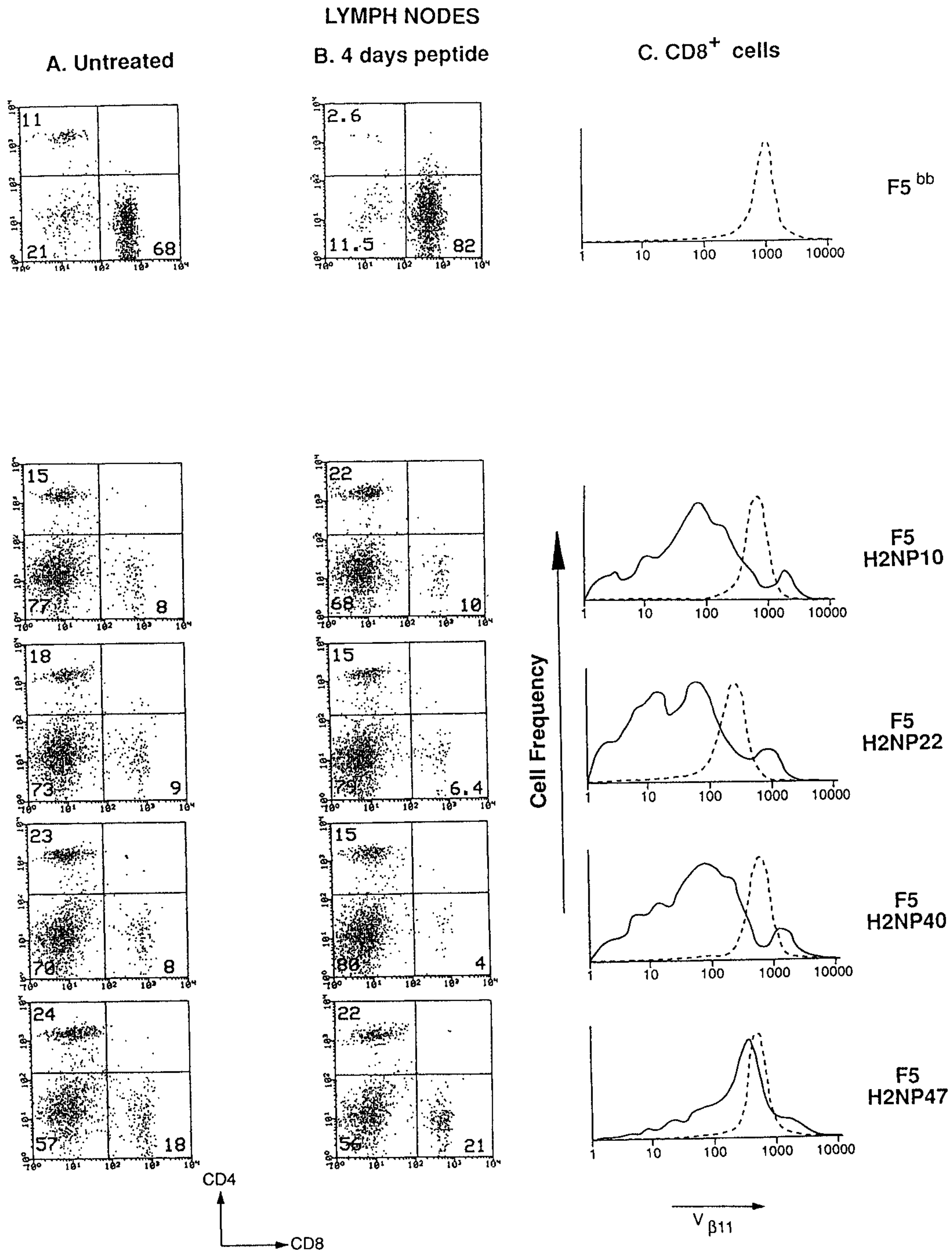

FIGURE 4. Double-transgenic mice have reduced numbers of CD8+ peripheral T cells with low levels of transgenic receptor. Three-color fluorometric analysis was performed on lymph node cells of TCR single-transgenic mice or TCR/NP double-transgenic mice before (A) or after (B) intraperitoneal treatment for 4 days with 50 n moles of antigenic peptide. Cells were stained with anti-CD8 FITC, anti-CD4 PE, and biotinylated anti-V$\beta_{11}$ followed by Tricolor-conjugated streptavidin as described in Materials and Methods. Dot blots represent two-color analysis of cells stained with CD4 and CD8. Numbers represent percent proportion of cells in the quadrant. (C) Three-parameter data files were software-gated to generate single-color staining histograms of V$\beta_{11}$ expression on CD8+CD4- single-positive cells. Numbers indicate the mean fluorescence of V$\beta_{11}$ staining in arbitrary units. Solid line: V$\beta_{11}$ expression on total thymocytes from double-transgenic mice. Dashed line: V$\beta_{11}$ expression on total thymocytes of the control F5 single-transgenic mouse.

1993a). When the $V\beta_{11}$ profile of total thymocytes from F5/NP double-transgenic mice was compared with that of F5 single-transgenic control mice, it became evident that the thymus from F5/H2NP10, F5/H2NP22, F5/H2NP40, and F5/H2NP47 mice was almost devoid of the $TCR^{hi}$ population (Fig. 3C).

The findings in the thymus were reflected in the periphery of F5/H2NP double-transgenic mice. Lymph nodes of the mice described before were stained for CD4, CD8, and $V\beta_{11}$. Figure 4A shows that the proportion of $CD8^+$ cells was drastically reduced from an average of 53–68% in F5 single-transgenic mice to 8–18% of lymph node cells in the various F5/NP double-transgenic mice. Gated $CD8^+$ cells were analyzed for expression of $V\beta_{11}$ and Fig. 4C shows such analysis. In all F5/NP double-transgenic mice, the circulating $CD8^+$ cells have reduced levels of transgenic TCR. However, this reduction varied from line to line, with F5/H2NP10 being most and F5/H2NP47 least affected. The majority of cells with high levels of $V\beta_{11}$ present in most double-transgenic mice probably represent cells expressing endogenous receptors as these are not as evident in mice that cannot rearrange endogenous TCR genes (see Figs 5 and 6).

Because allelic exclusion in TCR mice is not complete, particularly at the α-chain gene locus (Mamalaki et al., 1993b), the presence of double-positive $CD4^+8^+$ thymocytes and $CD8^+$ $V\beta_{11}^+$ peripheral cells in F5/NP double-transgenic mice could be explained by the fact that they may express endogenous α and β receptors, which allow them to be positive selected.

To test this possibility, we generated F5/NP double-transgenic mice unable to rearrange endogenous TCR genes. Thus, F5/H2NP mice were bred with Recombination Activating Gene-1–deficient mice ($RAG\text{-}1^{-/-}$) (Spanopoulou et al., 1994). Figure 5 shows that, in the absence of endogenous TCR rearrangement, $CD4^+8^+$ thymocytes and peripheral $CD8^+$ cells are still present in F5/H2NP22 mice. However, the $CD8^+$ T cells found in lymphoid organs are reduced in absolute numbers (not shown) and have lower levels of TCR and its coreceptor (CD8) in comparison with $CD8^+$ cells from F5/$RAG\text{-}1^{-/-}$ (Fig. 5).

The absence of endogenous receptors allowed us also to compare the levels of TCR on $CD8^+$ peripheral T cells in different double-transgenic mice. Figure 6 shows that F5/H2NP22/$RAG\text{-}1^{-/-}$ and F5/H2NP40/$RAG\text{-}1^{-/-}$ mice have considerably lower levels of T-cell receptor than F5/$RAG\text{-}1^{-/-}$ single-transgenic mice or F5/H2NP47/$RAG\text{-}1^{-/-}$ double-transgenic mice. These results indicate that to maintain tolerance, the levels of TCR adjust to the amount of antigen present and are consistent with the F5 T-cell proliferation studies shown in Fig. 2 that reflect variable antigen levels. Thus, mice expressing high levels of NP (H2NP40 or H2NP22) downregulate the F5 TCR to lower levels than mice with reduced expression of NP (H2NP47). The levels of TCR in F5/H2NP47 mice appear to be less affected in $RAG\text{-}1^{-/-}$ mice than in $RAG\text{-}1^{+/+}$ mice. The reason for this is under investigation at the moment. It is possible that unresponsiveness in the different double-transgenic mice is achieved by different mechanisms.

We conclude from these results that the tolerance in the double-transgenic mice is mainly due to the reduction in output of single-positive cells by arrest and/or deletion at the transition between the $CD4^+8^+TCR^{lo}$ to $CD4^+8^+TCR^{hi}$ stage. However, a small proportion of cells mature to the $CD8^+$ single-positive stage with low levels of TCR and are released in the periphery even in mice that cannot rearrange the endogenous TCR genes. No $CD4^-8^-$ $TCR^{hi}$ T cells were detected in any of the double-transgenic mice (not shown).

### CD44 Is Upregulated in $CD8^+$ from F5/H2NP Double-Transgenic Mice

CD44 is a marker whose upregulation has been taken as an indication that T cells have been exposed to cognate antigen (Budd et al., 1987). F5, F5/H2NP22, and F5/H2NP47 spleen cells were stained for CD44, CD8, and $V\beta_{11}$. $CD8^+$ $V\beta_{11}^+$ cells were gated and levels of CD44 on their surface were assessed. As seen in Fig. 7, CD44 is upregulated on CD8 T cells from double-transgenic mice in comparison with CD8 T cells from F5 mice. These findings indicate that these T cells have encountered nucleoprotein. Similar results were obtained in $RAG\text{-}1^{-/-}$/F5 excluding the possibility that these T cells express endogenous receptors that have responded to environmental antigens irrelevant to the F5 TCR (data not shown). These data are in agreement with studies that described increased levels of CD44 on tolerant T cells in transgenic mice expressing the cognate antigen (von Boehmer et al., 1991; Alferink et al., 1994).

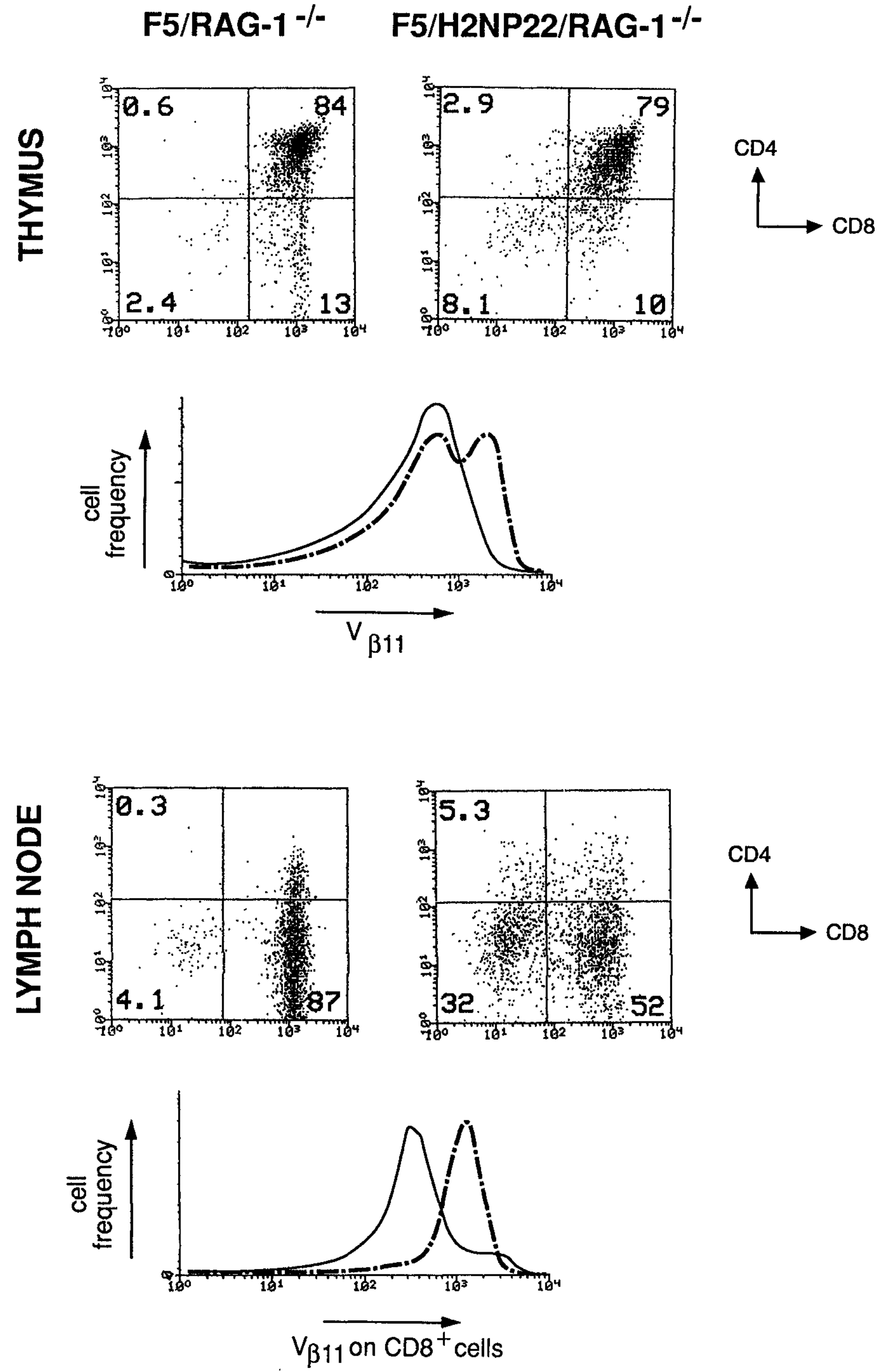

FIGURE 5. Presence of double-positive thymocytes and mature CD8+ cells in F5/H2NP/RAG-1–deficient double-transgenic mice. Three-color fluorometric analysis was performed on thymocytes or lymph node cells of TCR single-transgenic mice or TCR/NP double-transgenic mice bred onto RAG-1–deficient mice background. Cells were stained with anti-CD8 FITC, anti-CD4 PE, and biotinylated anti-V$\beta_{11}$ antibodies followed by Tricolor-conjugated streptavidin as described in Materials and Methods. Dot blots represent two-color analysis of cells stained with CD4 and CD8. Numbers represent percent proportion of cells in the quadrant. Three-parameters data files were software-gated to generate signle-color staining histograms of V$\beta_{11}$ on total thymocytes (thymus) or on CD8+ cells (lymph node). Numbers indicate the mean fluorescence of V$\beta_{11}$ staining in arbitrary units. Solid line: V$\beta_{11}$ expression on thymocytes or CD8+ lymph node cells from F5/H2NP22/RAG-1$^{-/-}$ double-transgenic mice. Dashed dotted line: V$\beta_{11}$ expression on total thymocytes of the control F5/RAG-1$^{-/-}$ single-transgenic mouse.

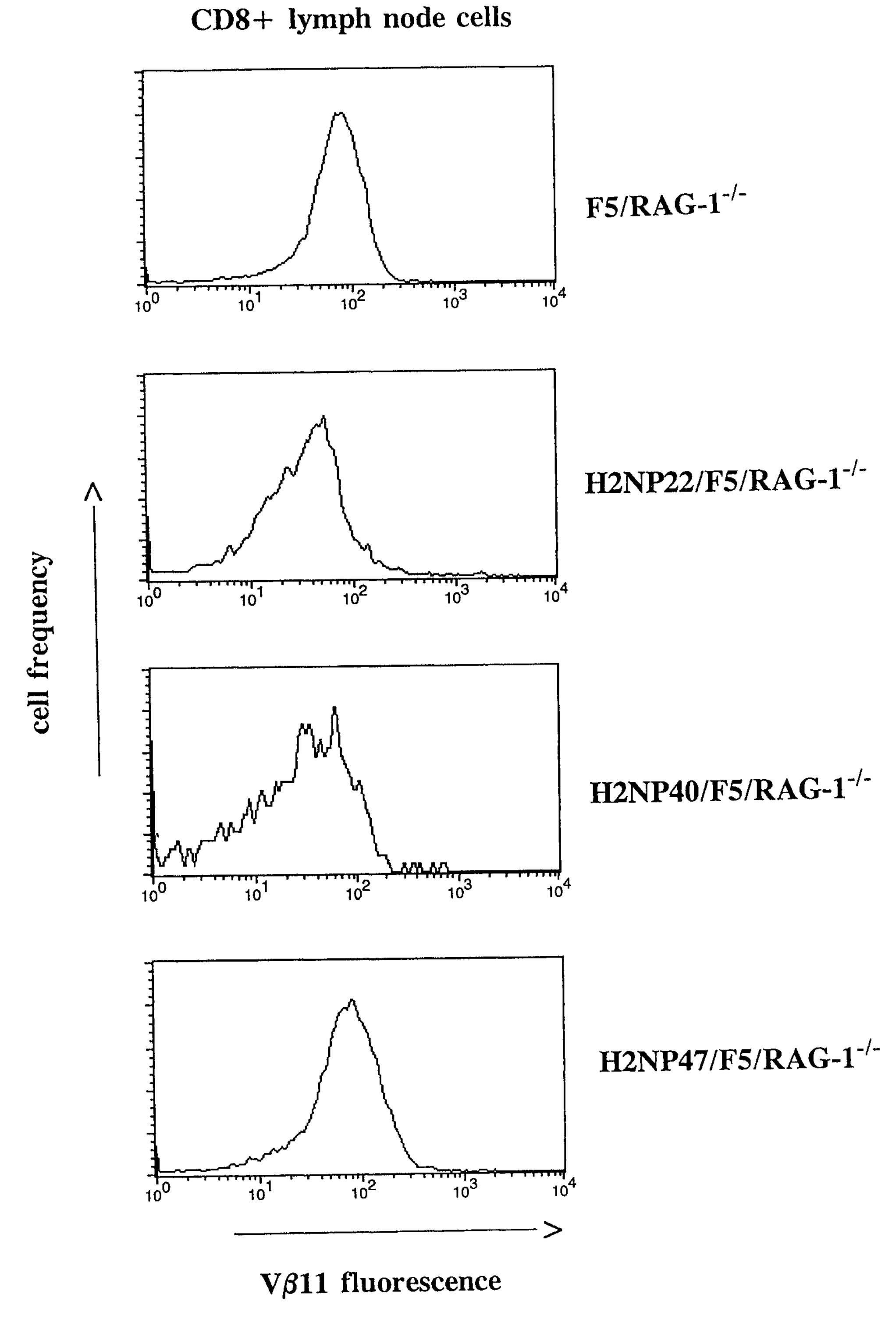

FIGURE 6. TCR levels on peripheral CD8+ T cells in F5/H2NP/RAG-1–deficient double-transgenic mice. Three-color fluorometric analysis was performed on lymph node cells of F5/RAG-1-/-, H2NP22/FR/RAG-1-/-, H2NP40/F5/RAG-1-/-, and H2NP47/F5/RAG-1-/- mice. Cells were stained with anti-CD8 FITC, anti-CD4 PE, and biotynalyted anti-V$\beta_{11}$ followed by Tricolor-conjugated streptavidin as described. Three-parameter data files were software-gated to generate single-color staining histograms of V$\beta_{11}$ on CD8+ cells.

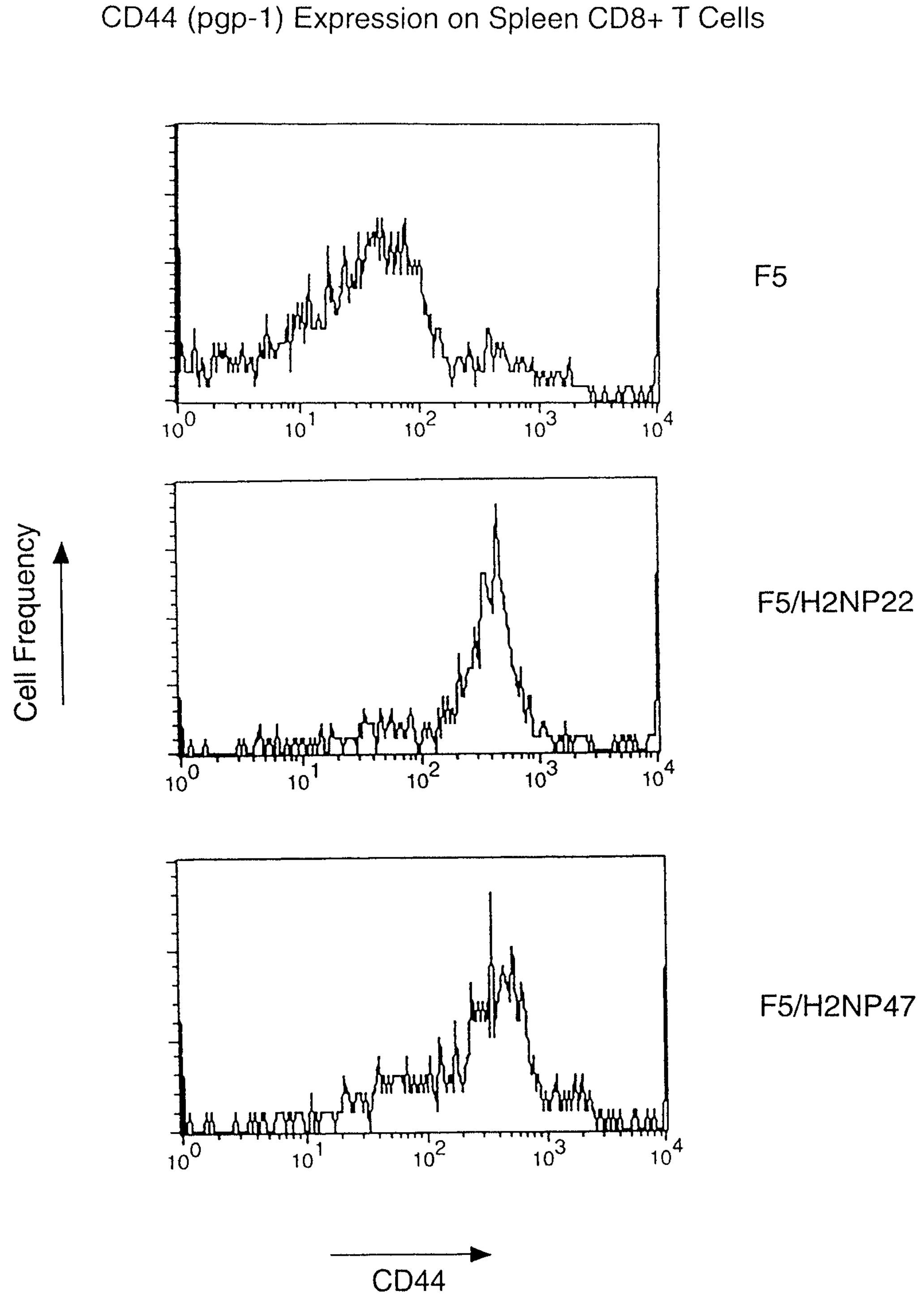

FIGURE 7. Peripheral CD8+ cells from F5/H2NP double-transgenic mice bear upregulated levels of the CD44 memory marker. Three-color fluorometric analysis was performed on spleen cells from F5 transgenic and F5/H2NP double-transgenic mice. Cells were stained with anti-CD8 PE, anti-V$\beta_{11}$ FITC, and biotinylated anti-CD44 followed by Streptavidin red. Histograms represent the levels of CD44 on CD8+ positive cells in F5, F5/H2NP22, and F5/H2NP47 spleens.

### Response of Double-Transgenic Mice to Additional Exposure to Antigen

F5 TCR-transgenic mice respond *in vivo* to four daily injections of 50 n moles of the antigenic peptide by proliferation of the $CD8^+$ T cells and by depletion of the double-positive $CD4^+8^+$ thymocytes (Mamalaki et al., 1992). To assess to what extent these phenomena can be seen in similarly treated double-transgenic mice, we administered 4 daily interperitoneal injections of 50 n moles of NP peptide.

Figure 3B shows representative two-color FACS analyses of thymocytes stained with CD4 and CD8 following 4 days of peptide administration. F5 single-transgenic control mice treated with peptide for 4 days show a marked decrease (10–30-fold) of thymus cellularity. In contrast, F5/H2NP10, F5/HPNP22, and F5/H2NP40 show a decrease in the number of thymocytes of approximately 2–3-fold (data not shown). The exception is double-transgenic mouse F5/H2NP47, which shows extensive thymic depletion similar to that seen in the F5 single-transgenic control mouse after peptide treatment. In the representative experiment shown in Fig. 3B, the reduction in the proportion of double-positive thymocytes in double-transgenic mice treated with peptide was from 71% to 62% in F5/H2NP10, from 73% to 56% in F5/H2NP22, from 74% to 62% in F5/H2NP40, and from 86% to 12% in F5/H2NP47 mice.

The proportion of $CD8^+$ T cells in the lymph nodes of F5/NP double-transgenic mice did not change upon exposure to antigenic peptide *in vivo* (Fig. 4B). As expected, no change was noted in the expression of $V\beta_{11}$ on gated $CD8^+$ lymph node cells from F5/H2NP10, F5/H2NP22, and F5/H2NP40 double-transgenic mice after administration of antigenic peptide (Fig. 8), presumably because most of these cells express endogenous $\alpha$ and/or $\beta$ receptors. However, when F5/H2NP22/RAG-$1^{-/-}$ were treated with peptide, the transgenic TCR on the few circulating $CD8^+$ was further downmodulated (Fig. 8). We concluded from these results that tolerance in most F5/NP double-transgenic mice involves the $CD8^+$ population and is sufficient to protect the mice from self-reactivity due to the low levels of TCR and coreceptor on these cells. However, this tolerance can be enhanced by exposure to higher levels of antigenic peptide.

### Functional Analysis of T Cells from Double-Transgenic Mice

To test the functional capability of peripheral T cells from F5/NP double-transgenic mice or from mice capable of presenting endogenous superantigen to the F5 TCR ($F5^{bk}$) (Mamalaki et al., 1993a), we assessed the proliferative response and the development of cytolytic activity by these cells after *in vivo* or *in vitro* stimulation with cognate peptide. Table 1 shows the results of exposing spleen cells from $F5^{bb}$, $F5^{bk}$, and F5/NP double-transgenic mice untreated or following peptide administration *in vivo* for 4 days, to H-$2^b$ splenic APC, alone or peptide-pulsed, in the presence of low concentrations of rIL-2 (2.5 IU/ml). In each of the two experiments, spleen cells from the $F5^{bb}$ control mice made good peptide-specific proliferative responses before and after 4 days *in vivo* peptide administration. In contrast, spleen cells from the $F5^{bk}$ heterozygotes not given peptide *in vivo* failed to make a peptide-specific proliferative response *in vitro*, although they did so following exposure to peptide *in vivo*. None of the F5/NP double-transgenic mice made peptide-specific proliferative responses, even after exposure to peptide *in vivo* (Table 1). Peptide-specific cytotoxic T cells were found *ex vivo* in spleen cells removed from $F5^{bb}$ and $F5^{bk}$ mice following peptide administration but never in untreated $F5^{bb}$ or $F5^{bk}$ mice, nor in any of the F5/NP double-transgenic mice, whether or not given peptide *in vivo* (data not shown). In contrast, spleen cells from all $F5^{bb}$, $F5^{bk}$, and F5/NP double-transgenic mice could develop peptide-specific cytotoxic effector cells after culture *in vitro* for 3 or 4 days in the presence of 20 IU rIL-2/ml. Table 2 shows the results of cytotoxicity developed after *in vitro* culture with rIL-2 and peptide-pulsed APC.

TABLE 1
Generation of Peptide-Specific Proliferative Responses *In Vitro* from Spleen Cells of F5 Mice Expressing Endogenous Superantigen or Cognate Peptide from a Transgene

| | | | $^3$H proliferation | |
|---|---|---|---|---|
| Experiment | Mouse | Treat$^p$ | +B10 | +B10$^p$ |
| 1 | F5(b) | — | 1,360 | 10,391 |
| | F5(b) | 4 dp | 1,721 | 64,417 |
| | F5(bk) | — | 2,131 | 3,058 |
| | F5(bk) | 4 dp | 2,829 | 10,638 |
| | F5/H2NP47 | — | 1,094 | 668 |
| | F5/H2NP47 | 4 dp | 1,391 | 974 |
| 2 | F5(b) | — | 422 | 15,994 |
| | F5(b) | 4 dp | 1,028 | 112,152 |
| | F5/H2NP40 | — | 1,676 | 1,690 |
| | F5/H2NP40 | — | 1,203 | 3,099 |
| | F5/H2NP40 | 4 dp | 4,557 | 4,311 |
| | F5/H2NP40 | 4 dp | 1,700 | 3,660 |

*Note*: Treat$^p$: *In vivo* treatment of mice with NP peptide. dp: Days with NP peptide. B10$^p$: Spleen cells from C57B1/10 mice loaded with NP peptide.

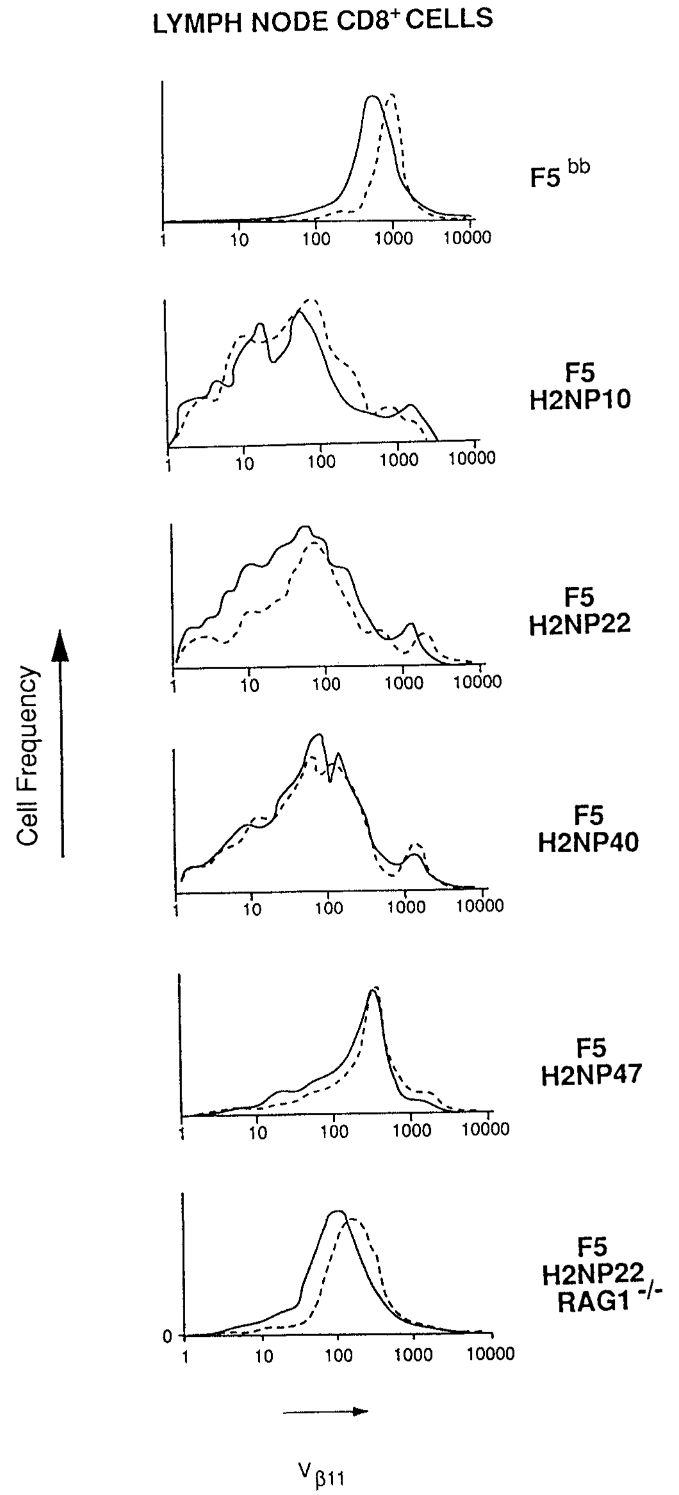

FIGURE 8. Levels of TCR on peripheral CD8+ T cells after treatment of double-transgenic mice with antigenic peptide. Three-parameter data files were software-gated to generate single-color staining histograms of $V\beta_{11}$ on $CD8^+CD4^-$ single-positive cells. Numbers indicate the mean fluorescence of $V\beta_{11}$ staining in arbitrary units. Solid line: $V\beta_{11}$ expression on gated $CD8^+$ lymph node cells from single- or double-transgenic mice before (dashed line) or after (solid line) intraperitoneal treatment for 4 days with 50 nM of antigenic peptide.

TABLE 2
Generation of Peptide-Specific Cytotoxicity *In Vitro* from Spleen Cells of F5 Mice Expressing Endogenous Superantigen or Cognate Peptide from a Transgene

| | | | Cytotoxicity | |
|---|---|---|---|---|
| Experiment | Mouse | Treat$^P$ | EL-4 | EL-4$^P$ |
| 1 | F5(b) | — | 0 | 13 |
| | F5(b) | 4 dp | 0 | 48 |
| | F5(bk) | — | 0 | 23 |
| | F5(bk) | — | 0 | 19 |
| | F5(bk) | 4 dp | 0 | 55 |
| | F5(bk) | 4 dp | 0 | 19 |
| 2 | F5(b) | — | 0 | 56 |
| | F5(b) | 4 dp | 0 | 71 |
| | F5/H2NP47 | — | 9 | 59 |
| | F5/H2NP47 | 4 dp | 10 | 46 |
| | F5/H2NP47 | 4 dp | 8 | 51 |
| 3 | F5(b) | — | 3 | 39 |
| | F5(b) | 4 dp | 1 | 52 |
| | F5/H2NP40 | — | 1 | 13 |
| | F5/H2NP40 | — | 1 | 22 |
| | F5/H2NP40 | 4 dp | 2 | 12 |
| | F5/H2NP40 | 4 dp | 2 | 20 |

*Note*: Treat$^P$: *In vivo* treatment of mice with NP peptide. dp: Days with NP peptide. EL-4$^P$: EL-4 cells loaded with NP peptide.

### DISCUSSION

Experiments using normal mice have shown that mature T cells reactive to endogenous superantigens are absent in adult mice and that whereas CD4$^+$8$^+$ double-positive thymocytes expressing the relevant TCR Vβ are present, such TCR Vβ$^+$ cells are absent or reduced in single-positive T cells in the thymus and the periphery (Kappler et al., 1987; MacDonald et al., 1988). Mice transgenic for antigen-specific T-cell receptors have extended these observations (Kisielow et al., 1988; Berg et al., 1989; Pircher et al., 1989; Hämmerling et al., 1991; Morahan et al., 1991; Schönrich et al., 1991; Auphan et al., 1992; Husbands et al., 1992; Geiger et al., 1993; Schönrich et al., 1993; Oehen et al., 1994; Sponaas et al., 1994).

In this paper, we studied the forms of negative selection and tolerance induction caused in F5 TCR-transgenic mice by nominal antigen when the latter is expressed in the form of a transgene. In the thymus of F5/NP double-transgenic mice, a complete absence of F5 TCR$^{hi}$ thymocytes is observed. In peripheral tissues, CD4$^+$ cells appear not to be affected in numbers or in the levels of V$\beta_{11}$ that they express. This is consistent with the probability that CD4$^+$ T cells in these mice are selected because they express endogenous receptors allowing their positive selection (Corbella et al., 1994). The CD8$^+$ peripheral mature population on the other hand is affected both in numbers and in the levels of V$\beta_{11}$ they express. In RAG-1$^{+/+}$ mice, the majority of these CD8$^+$ cells probably express endogenous α and/or β chains and this may be the reason they are positively selected and released in the periphery. It is possible that these cells, due to the presence of the antigen, downregulate the transgenic receptor, causing upregulation of the recombination machinery and consequently increased rearrangement of the endogenous TCR gene loci. This would be analogous to experiments describing receptor editing in self-reactive B cells (Gay et al., 1993; Tiegs et al., 1993).

In F5/RAG-1$^{-/-}$ mice, we still found CD8$^+$ mature T cells that are tolerant due to F5 TCR and CD8 coreceptor downregulation. We found a correlation between the extent of F5 TCR downregulation and the apparent levels of NP antigen expression on antigen-presenting cells in H2NP mice. That is, the more antigen, the lower the receptor. Therefore, it appears that T cells are capable of adjusting the expression of their antigen receptor to levels not capable of responding to the self-antigen. This has implications in our understanding of autoimmunity where T cells with potential autoreactive TCRs escape clonal deletion in the thymus and differentiate to mature T cells with lower levels of TCR. Such cells, when encountering foreign antigen of "better fit," may be activated and following TCR upregulation could attack cells expressing self cognate antigen.

An interesting observation concerns the reactivity of F5 CD8$^+$ T cells present in H2$^{bk}$ mice compared to those found in F5/H2NP double-transgenic mice. In both cases, CD8$^+$ T cells in peripheral lymphoid organs have low levels of TCR and are present in lower numbers. However, as we reported before (Mamalaki et al., 1993a; and this study), F5 CD8$^+$ cells from F5/H2$^{bk}$ mice that are presumably tolerant to endogenous Mtv antigens still react to cognate antigen (Table 1). On the other hand, F5 CD8$^+$ T cells from F5/H2NP mice only respond to cognate peptide by downregulating their TCR even further. This is a similar situation reported with mice transgenic for a TCR specific for LCMV and an endogenous superantigen (Pircher et al., 1989; Kawai and Ohashi, 1995).

We conclude from these findings that in F5TCR mice, negative selection of self-reactive thymocytes, whether to H-2E/Mtv (MHC Class II restricted reactivity) or to nucleoprotein (MHC Class I reactivity), occurs at the transition from CD4$^+$8$^+$TCR$^{lo}$ to CD4$^+$8$^+$TCR$^{hi}$. It is unclear at the moment whether this involves physical elimination of developing thymocytes at the point of upregulation of the receptor or arrest of these thymocytes at the TCR$^{lo}$ stage due to the presence of the antigen (Takahama et al., 1992; Swat et al., 1994).

The picture observed in F5/NP double-transgenic mice strongly resembles that developing in F5 single-transgenic mice treated with cognate peptide long term. In such long-term peptide-treated mice, the thymus contains CD4$^{+}$8$^{+}$ cells bearing low levels of TCR and the peripheral lymphoid organs contain very few CD8$^{+}$ T cells with low levels of transgenic TCR (Mamalaki et al., 1993b). Thus, chronic exposure of mice to cognate antigen leads to a situation similar to tolerance toward self-antigens.

Additional exposure of tolerant T cells to high levels of antigen in other experimental models has led to further tolerization of these cells by downmodulation of the TCR and/or the coreceptor (Hämmerling et al., 1991). Further antigenic exposure of T cells from F5/NP double-transgenic mice did not enhance their tolerization, judging from the levels of TCR and coreceptor: These remained unchanged after treatment with the antigenic peptide. When we repeated this experiment using RAG-1$^{-/-}$ mice, we saw that the few CD8$^{+}$ F5TCR$^{lo}$ cells present in the periphery did downregulate their receptor and coreceptor even further upon exposure to higher levels of antigen given as exogenously administered peptide. This is in agreement with evidence from an alloreactive transgenic TCR model (Hämmerling et al., 1991), and confirms that whereas the tolerance induced in double-transgenic mice is sufficient for the levels of nucleoprotein transgene expressed, however, when higher levels of antigen are introduced, the cells downregulate their TCR even further.

Despite unresponsiveness *in vivo*, stimulation of T cells from double-transgenic mice was possible *in vitro*. Peripheral splenic T cells from all F5 mice, whether expressing a "deleting" endogenous superantigen or a cognate peptide-generating transgene, can generate peptide-specific cytotoxicity after *in vitro* culture 72–96 hr in the presence of moderate doses (20 IU/ml) of rIL-2. This shows that the apparently anergic phenotype manifest *in vivo* can be converted to activation in the presence of IL-2. This is one of the hallmarks of anergic T cells and perhaps should not surprise us. On the other hand, the observation that the proliferative capacity of these anergic T cells is severely compromised is an interesting one, suggesting that whereas terminal differentiation into effector cells is still possible, clonal expansion is not. The F5/NP double-transgenic mice cannot be distinguished from each other in this respect, but the F5$^{bk}$ mice are different in that their spleen cells following *in vivo* exposure to exogenous cognate peptide will subsequently clonally expand on *in vitro* stimulation with peptide-pulsed APC. The extent to which F5 T cells are restrained *in vivo* in the continuous presence of potentially activating antigen is a measure of the capacity of the organism to control autoreactivity. The fail-safe mechanisms could clearly involve downregulation of TCR and/or accessory molecules, as documented in this paper, but the ubiquitous presence of antigen on nonprofessional APC cells in the periphery also could have an inhibitory effect on the activation cascade via molecular interactions at present poorly understood.

In a study by Oehen et al. (1994), it was shown that in double-transgenic mice for LCMV glycoprotein and a TCR specific for this antigen, when the antigen is expressed at low levels, transgenic TCR$^{+}$ T cells are partially deleted. However, these T cells retain their capacity to proliferate and mount a cytolytic response. In our system of double-transgenic mice with even the lowest levels of antigen expression, TCR$^{+}$ T cells do not respond *in vivo*, do not proliferate when stimulated by antigen *in vitro*, and develop cytolytic activity only after culture *in vitro* with IL-2. These differences may reflect variations in TCR/MHC-Ag affinities.

Results in this and previously published papers indicate that negative selection in the thymus can take place at different stages of thymocyte development with the ultimate result the absence of mature TCR$^{hi}$ self-reactive T cells. This can be accomplished as early as during the transition from CD4$^{-}$8$^{-}$TCR$^{-}$ to CD4$^{+}$8$^{+}$TCR$^{lo}$ (H-Y-, LCMV, MHC-specific TCRs) or later during the transition from CD4$^{+}$8$^{+}$TCR$^{lo}$ to CD4$^{+}$8$^{+}$TCR$^{hi}$ (H-2E$^{+}$Mtv$^{+}$-specific TCRs, F5/H2NP).

The actual stage in development in which deletion of self-reactive clones or the establishment of their unresponsiveness occurs is most likely dependent on the affinity of the TCR for the MHC/peptide ligand, on the site of expression of the deleting ligand (cortex/medulla), the type of cells that express the deleting ligand, and finally the levels of this expression.

### MATERIALS AND METHODS

### Mice and Gene Constructs

Mice were generated and maintained in a conventional colony free of pathogens at the National Institute for Medical Research in London, F5 TCR and H2NP transgenic mice were generated as described previously (Lang et al., 1988; Mamalaki et al., 1993a) using inbred C57B1/10 mice. Influenza nucleoprotein peptide (αα366–374) was dissolved in PBS and injected intraperitoneally as indicated in the figure legends.

The H2NP fragment for microinjection was constructed by combining fragments containing the H2-$K^b$ promoter, the influenza nucleoprotein coding sequences, and the SV40 splice and polyadenylation signal in three steps: First, a BamHI–SmaI fragment from the pBG311 expression vector (Cate et al., 1986) was cloned in the BamHI–SmaI sites of the polylinker of Bluescript (Stratagene). This fragment contains the SV40 small-t-antigen gene-splice sequence, the large-T-antigen gene-polyadenylation sequence, and part of a polylinker including SmaI-, NdeI, SstI, and BglII-restriction sites. Second, in the BglII site of this polylinker was inserted a BamHI fragment from the PUC9/IMP1295 plasmid (Davey et al., 1985; Townsend et al., 1985) that contains sequences coding for αα1, 2, and 328–498 of the nucleoprotein from the influenza virus (the deletion removes amino acids 3–327). Third, the resulting plasmid was cleaved at the EcoRI site of the Bluescript polylinker and the SmaI site described in the first cloning step and an EcoRI-NruI fragment from the H2-$K^b$ promoter was inserted (Weiss et al., 1983). The microinjection fragment was isolated free from vector sequences by digesting with EcoRI and NotI.

### Flow Cytometry

For three-color analysis, $10^6$ thymocytes or lymph node cells were stained with the following antibodies in different combinations: phycoerythrin-conjugated anti-CD4 (GK1.5) (Becton Dickinson), fluorescein isothiocyanate-conjugated anti-CD8 (53–6.7) (Becton Dickinson), phycoerythrin-conjugated anti-CD8 (YTS 169.4) (Coulter Immunology), biotinylated anti-V$\beta_{11}$ (KT11) (Tomonari and Lovering, 1988) and biotinylated anti-CD44 (pgp-1) (kind gift from Dr Stockinger), followed by a second layer of Tricolor-conjugated streptavidin (Caltag) or streptavidin red 670 (Gibco). Three-color FACS analysis was performed with a FACScan laser instrument and Lysis I or II program (Becton Dickinson).

### Isolation of Antigen in Presenting Cells

Antigen-presenting cells were prepared by a modified protocol published previously (Tanaka, 1993). Thymuses and spleens from adult B10, NP40, and NP47 mice were teased gently and placed in a cocktail with collagenase (Worthington, Biochemical Corp., Freehold, NJ; 1.6 mg/ml) and DNAse (Sigma, 0.1%) in RPMI for 1 hr at 37°C. The cells were washed, resuspended, and plated in RPMI 10% FCS. Contaminating red cells were removed by hypotonic shock. After 2 hr incubation, the nonadherent cells were washed off and adherent ones were cultured overnight at 37°C. After 18 hr, the dendritic cells were recovered as the nonadherent population. To minimize the contamination with thymocytes and T cells, the pooled cells were washed with PBS and treated with 5 mM EDTA for 10 min at room temperature. The adherent cells were identified as macrophages by their distinctive morphology and were treated with trypsin/EDTA for 30 min, washed twice, resuspended, and plated in RPMI 10% FCS. Cells were sensitized with NP366–374 by adding 20 μM/ml of peptide for 1 hr at 37°C, then washed twice in medium, and resuspended.

### Cytotoxic T-cell Assays

These were carried out using as targets EL-4 cells growing in log phase *in vitro*: Two aliquots of $2 \times 10^6$ EL-4 were labeled for 60 min at 37°C in 0.1 ml BSS medium containing 100 μCi $^{51}$Cr sodium chromate; to one aliquote, 100 μl of 100 μM influenza nucleoprotein peptide (NP366–374) was also added at the beginning of the 60-min incubation. After incubation, both aliquots of EL-4 were washed twice and then resuspended in complete RPMI medium at $1 \times 10^5$/ml before dispensing 100 μl ($1 \times 10^4$) into each microtitre well into which serial dilutions of effector T cells had been placed.

Effector cells generated in MLC from spleen cells of transgenic mice, cultured at $2 \times 10^6$ cells per 2-ml well at 37°C in a 5% $CO_2$ atmosphere for 3 to 5 days in the presence of 25 IU/ml human rIL-2 with or without $5 \times 10^6$ irradiated cells per well of C57BL/10 spleen cells sensitized or not with NP366–374 peptide, as described before. Effector cells were harvested, centrifuged, and resuspended in fresh complete RPMI medium, counted, and then volumes adjusted to give concentration of $3 \times 10^6$/ml. Each effector cell suspension was dispensed in round-bottomed microtitre wells in a volume of 100 µl in triplicate and then four serial, one in three dilutions were carried out. After addition of $1 \times 10^4$ target cells/well, effector-to-target-cell ratios of 30:1, 10:1, 3:1, and 1:1 were present. The assay plates were briefly centrifuged then incubated for 3 hr at 37°C in a 5% $CO_2$ atmosphere. One hundred microliters of supernatant was then harvested from each plate and counted for gamma rays. The percent specific lysis was calculated according to the formula

$$\% \text{ specific lysis} = \frac{E - C}{M - C} \times 100$$

where $E$ is cpm from wells with effectors present, $C$ is the cpm from control wells with target cells incubated in medium alone, and $M$ is the maximum released counts from target cells incubated with 5% Triton. Twelve-point regression analysis was performed for each titration curve and the percent lysis at an effector: target ratio of 10:1 taken from this curve. Significant positive lysis was taken as levels over 10% specific lysis from curves where the $r^2$ value lay between 0.80 and 1.00.

### Proliferation Assays

Responder cells were suspensions of spleen cells in RPMI medium supplemented with 10% FCS, $5 \times 10^{-5}$ M 2 mercapto-ethanol, 100 IU/ml penicillin, 100 µg/ml streptomycin, 10 mM Hepes, and 2 mM glutamine. They were dispensed at $1 \times 10^4$ cells per 0.2 ml flat-bottomed microtitre well for proliferative MLR cultures. Human rIL-2 was added to make a final concentration of 10 IU/ml for proliferative assays. Cells used as a source of antigen were spleen-cell suspensions from which red blood cells had been removed by brief exposure to hypotonic shock. B10 spleen cells were used either alone (B10) or after 45 min incubation with 100 µM peptide (NP365–379) followed by two washes in RPMI ($B10^P$). Cells were subsequently irradiated 2500R from a $^{60}Co$ source immediately before addition to cultures: $5 \times 10^5$ antigen cells were added to each 0.2 ml microtitre well for the proliferation assay. Microtitre wells were pulsed at 72 hr with 1 µCi/well $^3H$ thymidine and harvested 6 hr later for beta scintillation counting.

### ACKNOWLEDGMENTS

The authors wish to thank Mrs Burke for expert secretarial assistance and Drs A. Mellor, B. Stockinger, T. Zal, and R. Zamoyska for scientific discussions and reagents. M.M. was supported by a grant from the Leukaemia Research Fund.

*(Received July 7, 1995)*

*(Accepted September 6, 1995)*

### REFERENCES

Alferink J., Schittek B., Schönrich G., Hämmerling G.J., and Arnold B. (1994). Long life span of tolerant T cells and the role of antigen in maintenance of peripheral tolerance. Int. Immunol. **7**: 331–336.

Auphan N., Schönrich G., Malissen M., Barad M., Hämmerling G., Arnold B., Malissen B., and Schmitt-Verhulst A.-M. (1992). Influence of antigen density on degree of clonal deletion in T cell receptor transgenic mice. Int. Immunol. **4**: 541–547.

Berg L.J., Fazekas de St Groth B., Pullen A.M., and Davis M.M. (1989). Phenotypic differences between αβ versus β T cell receptor transgenic mice undergoing negative selection. Nature **340**: 559–562.

Blackman M., Kappler J., and Marrack P. (1990). The role of the T cell receptor in positive and negative selection of developing T cells. Science **248**: 1335–1341.

Budd R.C., Cerottini J.-C., Norvath C., Bron C., Pedrazzini T., Howe R.C., and MacDonald H.R. (1987). Distinction of virgin and memory T lymphocytes. Stable acquisition of the Pgp-1 glycoprotein concomitant with antigenic stimulation. J. Immunol. **138**: 3120.

Cate R.L., Mattaliano R.J., Hession C., Tizard R., Farber N.M., Cheung A., Ninfa E.G., Frey A.Z., Gash D.J., Chow E.P., Fisher R.A., Bertonis J.M., Torres G., Wallner B.P., Ramachandran K.L., Ragin R.C., Manganaro T.F., MacLaughlin D.T., and Donahoe P.K. (1986). Isolation of the bovine and human genes for Mullerion inhibiting substance and expression of the human gene in animal cells. Cell **45**: 685.

Corebella P., Moskophidis D., Spanopoulou E., Mamalaki C., Tolaini M., Itano A., Lans D., Baltimore D., Robey E., and Kioussis D. (1994). Functional commitment to helper T cell lineage precedes positive selection and is independent of T cell receptor MHC specificity. Immunity **1**: 269–276.

Davy J., Dimmock N.J., and Colman A. (1985). Identification of the sequence responsible for the nuclear accumulation of the influenza virus nucleoprotein in xenopus oocytes. Cell **40**: 667–675.

Dyson P.J., Knigt A.M., Fairchild S., Simpson E., and Tomonari K. (1991). Genes encoding ligands for deletion of Vb11 T cells cosegragate with mammary tumour virus genomes. Nature **349**: 531.

Gay G., Saunders T., Camper S., and Weigert M. (1993). Receptor editing: An approach by autoreactive B cells to escape tolerance. J. Exp. Med. **177**: 999–1008.

Geiger T., Soldevilla G., and Flavell R.A. (1993). T cells are responsive to the simian virus 40 large tumor antigen transgenically expressed in pancreatic islets. J. Immunol. **151**: 7030–7037.

Hämmerling G.J., Schönrich G., Momburg F., Auphan N., Malissen M., Malissen B., Schmitt-Verhulst A.-M., and Arnold

B. (1991). Non-deletional mechanisms of peripheral and central tolerance: Studies with transgenic mice with tissue specific expression of a foreign MHC Class I antigen. Immunol. Rev. **122**: 47–67.

Husbands S.D., Schönrich G., Arnold B., Chandler P.R., Simpson E., Philpott K.L., Tomlinson P., O'Reilly L., Cooke A., and Mellor A.L. (1992). Expression of major histocompatibility complex class I antigens at low levels in the thymus induces T cell tolerance via a non-deletional mechanism. Eur. J. Immunol. **22**: 2655–2661.

Jones L.A., Chin L.T., Longo D.L., and Kruisbeek A.M., (1990). Peripheral clonal elimination of functional T cells. Science **250**: 1726–1729.

Kappler J.W., Roehm N., and Marrack, P. (1987). T-cell tolerance by clonal elimination in the thymus. Cell **49**: 273.

Kawabe Y., and Ochi A. (1991). Programmed cell death and extrathymic reductionn of Vβ8$^+$CD4$^+$ T cells in mice tolerant to *staphylococcus aureus* enterotoxin B. Nature **349**: 245–248.

Kawai K., and Ohashi P. (1995). Immunological function of a defined T-cell population tolerized to low-affinity self antigens. Nature **374**: 68–69.

Kisielow P., Bluthmann H., Staerz U.D., Steinmetz M., and von Boehmer H. (1988). Tolerance in T cell receptor transgenic mice involves deletion of nonmature CD4$^+$8$^+$ thymocytes. Nature **333**: 742–746.

Lang G., Wotton D., Owen M.J., Sewell W.A., Brown M.H., Mason D.Y., Crumpton M.J., and Kioussis D. (1988). The structure of the human CD2 gene and its expression in transgenic mice. EMBO J. **7**: 1675–1682.

MacDonald H.R., Schneider R., Lees R.K., Howe R.C., Acha-Orbea H., Festenstein H., Zingernagel R.M., and Hengartner H. (1988). T-cell receptor Vβ use predicts reactivity and tolerance to Mls$^a$-encoded antigens. Nature **332**: 40–45.

Mamalaki C., Elliott T., Norton T., Yannoutsos N., Townsend A.R., Chandler P., Simpson E., and Kioussis D. (1993a). Positive and negative selection in transgenic mice expressing a T-cell receptor specific for influenza nucleoprotein and endogenous superantigen. Dev. Immunol. **3**: 159–174.

Mamalaki C., Norton T., Tanaka Y., Townsend A.R., Chandler P., Simpson E., and Kioussis D. (1992). Thymic depletion and peripheral activation of class I major histocompatibility complex-restricted T cells by soluble peptide in T-cell receptor transgenic mice. Proc. Natl Acad. Sci. USA **89**: 11342–11346.

Mamalaki C., Tanaka Y., Corbella P., Chandler P., Simpson E., and Kioussis D. (1993b). T cell deletion follows chronic antigen specific T cell activation *in vivo*. Int. Immunol. **5**: 1285–1292.

Morahan G., Hoffman M.W., and Miller J.F.A.P. (1991). A nondeletional mechanism of peripheral tolerance in T-cell receptor transgenic mice. Proc. Natl Acad. Sci. USA **88**: 11421–11425.

Mueller D.L., Jenkins M.K., and Schwartz R.H. (1989). Clonal expansion versus functional clonal inactivation: A costimulatory signalling pathway determines the outcome of T cell antigen receptor occupancy. Ann. Rev. Immunol. **7**: 445–480.

Oehen S.U., Ohashi P.S., Burki K., Hengartner H., Zinkernagel R.M., and Aichele P. (1994). Escape of thymocytes and mature T cells from clonal deletion due to limiting tolerogen expression levels. Cell Immunol. **158**: 342–352.

Pircher H., Burki K., Lang R., Hengartner H., and Zingernagel R.M. (1989). Tolerance induction in double specific receptor transgenic mice varies with antigen. Nature **342**: 559.

Ramsdell F. and Fowlkes B.J. (1990). Clonal deletion versus clonal anergy: The role of the thymus in inducing self tolerance. Science **248**: 1342–1348.

Rocha B., Vassalli P., and Guy-Grand D. (1992). The extrathymic T cell development pathway. Immunol. Today **13**: 449–458.

Schönrich G., Kallinke U., Momburg F., Malissen M., Schmitt-Verhulst A.M., Malissen B., Hämmerling G.J., and Arnold B. (1991). Down-regulation of T cell receptors on self-reactive T cells as a novel mechanism for extrathymic tolerance induction. Cell **65**: 293–304.

Schönrich G., Strauss G., Muller K.-P., Dustin L., Loh D.Y., Auphan N., Schmitt-Verhulst A.-M., Arnold B., and Hämmerling G.J. (1993). Distinct requirements of positive and negative selection for selecting cell type and CD8 interaction. J. Immunol. **151**: 4098–4105.

Schwartz R.H. (1989). Acquisition of immunologic self-tolerance. Cell **57**: 1073–1081.

Spanopoulou E., Roman C.A.J., Corcoran L.M., Schlissel M.S., Silver D.P., Nemazee D., Nussenzweig M.C., Shinton S.A., Hardy R.R., and Baltimore D. (1994). Functional immunoglobulin transgenes guide ordered B-cell differentiation in Rag-1-deficient mice. Genes Dev. **8**: 1030–1042.

Sponaas A.-M., Tomlinson P.D., Antoniou J., Auphan N., Langlet C., Malissen B., Schmitt-Verhulst A.-M., and Mellor A.L. (1994). Induction of tolerance to self MHC class I molecules expressed under the control of milk protein or β–globin gene promoters. Int. Immunol. **6**: 277–287.

Swat W., von Boehmer H., and Kisielow P. (1994). Central tolerance: Clonal deletion or clonal arrest? Eur. J. Immunol. **24**: 485–487.

Takahama Y., Shores E.W., and Singer A. (1992). Negative selection of precursor thymocytes before their differentiation into CD4$^+$CD8$^+$ cells. Science **258**: 653–656.

Tanaka Y., Mamalaki C., Stockinger B., and Kioussis D. (1993). *In vitro* vegative selection of αβ T cell receptor transgenic thymocytes by conditionally immortalized thymic cortical epithelial cell lines and dendritic cells. Eur. J. Immunol. **23**: 2614–2621.

Tiegs S., Russell D.M., and Nemazee D. (1993). Receptor editing in self-reactive bone marrow B cells. J. Exp. Med. **177**: 1009–1020.

Tomonari K., and Lovering E. (1988). T-cell receptor specific antibodies against a Vβ11-positive mouse T-cell clone. Immunogenetics **28**: 445–451.

Townsend A.R.M., Gotch F.M., and Davey J. (1985). Cytotoxic T cells recognise fragments of the influenza nucleoprotein. Cell **42**: 457.

Townsend A.R.M., Rothbard J., Gotch F.M., Bahadur G., Wraith D., and McMichael A.J. (1986). The epitopes of influenza nucleoprotein recognized by cytotoxic lymphocytes can be defined with short synthetic peptides. Cell **44**: 959–968.

von Boehmer H., Kirberg J. and Rocha B. (1991). An unusual lineage of α/β T cells that contains autoreactive cells. J. Exp. Med. **174**: 1001–1008.

Webb S., Morris C., and Sprent J. (1990). Extrathymic tolerance of mature T cells: Clonal elimination as a consequence of immunity. Cell **63**: 1249–1256.

Weiss E., Golden L., Zakut R., Mellor A., Fahrner K., Kvist S., and Flavell R.A. (1983). The DNA sequence of the H-2Kb gene: Evidence for gene conversion as a mechanism for the generation of polymorphism in histocompatibility antigens. EMBO J. **2**: 453.

